# Transcriptome-wide responses of adult melon thrips (*Thrips palmi*) associated with capsicum chlorosis virus infection

**DOI:** 10.1371/journal.pone.0208538

**Published:** 2018-12-07

**Authors:** Shirani M. K. Widana Gamage, Dorith Rotenberg, Derek J. Schneweis, Chi-Wei Tsai, Ralf G. Dietzgen

**Affiliations:** 1 Queensland Alliance for Agriculture and Food Innovation, The University of Queensland, St. Lucia, Queensland, Australia; 2 Department of Entomology and Plant Pathology, North Carolina State University, Raleigh, NC, United States of America; 3 Department of Plant Pathology, Kansas State University, Manhattan, KS, United States of America; 4 Department of Entomology, National Taiwan University, Taipei, Taiwan; Chinese Academy of Agricultural Sciences Institute of Plant Protection, CHINA

## Abstract

*Thrips palmi* is a widely distributed major agricultural pest in the tropics and subtropics, causing significant losses in cucurbit and solanaceous crops through feeding damage and transmission of tospoviruses. *Thrips palmi* is a vector of capsicum chlorosis virus (CaCV) in Australia. The present understanding of transmission biology and potential effects of CaCV on *T*. *palmi* is limited. To gain insights into molecular responses to CaCV infection, we performed RNA-Seq to identify thrips transcripts that are differentially-abundant during virus infection of adults. *De-novo* assembly of the transcriptome generated from whole bodies of *T*. *palmi* adults generated 166,445 contigs, of which ~24% contained a predicted open reading frame. We identified 1,389 differentially-expressed (DE) transcripts, with comparable numbers up- (708) and down-regulated (681) in virus-exposed thrips compared to non-exposed thrips. Approximately 59% of these DE transcripts had significant matches to NCBI non-redundant proteins (Blastx) and Blast2GO identified provisional functional categories among the up-regulated transcripts in virus-exposed thrips including innate immune response-related genes, salivary gland and/or gut-associated genes and vitellogenin genes. The majority of the immune-related proteins are known to serve functions in lysosome activity and melanisation in insects. Most of the up-regulated oral and extra-oral digestion-associated genes appear to be involved in digestion of proteins, lipids and plant cell wall components which may indirectly enhance the likelihood or frequency of virus transmission or may be involved in the regulation of host defence responses. Most of the down-regulated transcripts fell into the gene ontology functional category of ‘structural constituent of cuticle’. Comparison to DE genes responsive to tomato spotted wilt virus in *Frankliniella occidentalis* indicates conservation of some thrips molecular responses to infection by different tospoviruses. This study assembled the first transcriptome in the genus *Thrips* and provides important data to broaden our understanding of networks of molecular interactions between thrips and tospoviruses.

## Introduction

Thrips belong to the family Thripidae in the order Thysanoptera which contains nearly 7700 described thrips species [1]. However, less than 1% of them are considered as agricultural pests that cause crop damage directly by feeding and indirectly by transmitting tospoviruses [2]. At present, 15 thrips species have been reported to transmit tospoviruses [3]. Among them, *Frankliniella occidentalis* is world-wide the most devastating invasive species, with a broad host range, transmitting multiple tospoviruses (genus *Orthotospovirus*, family *Tospoviridae*, Order *Bunyavirales*) including the economically important tomato spotted wilt virus (TSWV) [4]. Melon thrips (*Thrips palmi*) originated in Southeast Asia [5] and have become a serious invasive pest in tropical and subtropical countries [6]. Several tospoviruses are known to be transmitted by *T*. *palmi* including calla lily chlorotic spot virus [7], groundnut bud necrosis virus [8], melon yellow spot virus [9], tomato necrotic ringspot virus [10], watermelon bud necrosis virus [11] and watermelon silver mottle virus [12]. In Australia, capsicum chlorosis virus (CaCV) is transmitted by *T*. *palmi* [13].

Thrips transmit tospoviruses in a persistent and propagative mode by which virus circulates and replicates within the thrips body [3]. Thrips acquire virus while feeding on infected plant tissues—most efficiently as first instar larvae–and the virus is retained during larval and pupal molts [14]. While viruliferous late second instar larvae can inoculate plants, adults are vector-competent only if the virus was acquired during the larval stages [15]. After ingestion, virions travel through the esophagus to the midgut—the primary site of virus entry—where they replicate and then disseminate and replicate in the surrounding visceral muscle tissue [16]. Virus also replicates in the primary salivary glands (PSG) of thrips [17]. Virus is then transmitted from salivary glands to plants during thrips feeding. Until recently, there was no evidence to indicate the exact infection route of TSWV from midgut to PSG. However, a recent study revealed progression of TSWV infection in larvae of *F*. *occidentalis* spread from midgut to ligaments and tubular salivary glands (TSG), where efferent salivary duct and filament structures connect TSG and PGS [18]. These authors further showed that during thrips development, the primary site of tospovirus replication shifts from midgut and TSG in larvae to PSG in adult thrips.

Tospoviruses have been shown to alter thrips vector performance and behavior both directly and indirectly. Direct negative effects on thrips reproductive potential and developmental time have been reported from TSWV-*F*. *fusca* [19, 20] and impatiens necrotic spot virus-*F*. *occidentalis* [21] interactions, however experimental evidence indicates no apparent negative effect of TSWV infection on life history traits of *F*. *occidentalis* [22, 23] or watermelon silver mottle virus on *T*. *palmi* [24]. Effects of TSWV infection on *F*. *occidentalis* have been documented, including enhanced reproduction [25], reduced developmental time [26, 27], and altered feeding behaviors [28]. Predictive models developed to study dynamics in virus spread suggest that TSWV infection may change thrips preferential feeding behavior and enhance survival [29]. Indirect effects include plant-mediated effects of virus infection on the performance, development, fecundity, survival and host preference of thrips vectors [3]. In general, the majority of tospovirus-thrips interactions report no apparent negative effects on the fitness of the vector. One hypothesis is that thrips mount molecular defense responses against virus infection that minimize cytopathological effects that could, if unharnessed, negatively impact their development and survival.

Recently, transcriptomes of two *Frankliniella* species, *F*. *occidentalis* and *F*. *fusca*, in response to tospovirus infection were reported [30, 31]. Both studies analysed larval, pupal and adult stages for whole-body responses to TSWV infection using high throughput sequencing (RNA-Seq). Gene ontologies that infer processes and functions associated with host defence, insect cuticle structure and development, metabolism and transport were affected by TSWV infection in *F*. *occidentalis* [30]. In *F*. *fusca*, TSWV-responsive genes were similarly associated with intracellular transport, development and immune responses [31]. Furthermore, the repertoire of responsive genes varied between developmental stages in both systems. In this study, we aimed to investigate a different thrips-tospovirus system involving the genus *Thrips* for the first time, to broaden our understanding of the molecular responses of thrips vectors exposed to tospovirus infection. We identified transcriptome-wide responses of *T*. *palmi* to CaCV infection, some of which were conserved in other thrips species in response to infection with different tospoviruses. This knowledge may be useful in future studies to identify molecular targets to interfere with tospovirus transmission by thrips.

## Materials and methods

### Maintenance of *T*. *palmi* colonies

A *T*. *palmi* colony derived from a pure culture maintained at the Vector Laboratory, National Taiwan University, Taipei, Taiwan was reared on bean (*Phaseolus coccineus*) seedlings following conditions previously established [24]. Oviposition of female thrips was enhanced by allowing them to feed on pollen (Hung Gee, Taiwan) in a sealed Petri plate containing a bean leaf. Cohorts of L1 larvae were transferred into a 2-L beaker enclosed with a fresh bean seedling and reared until adulthood in a growth cabinet at 25°C with 70% relative humidity and 16 h/8 h light/dark photoperiod.

To generate populations of CaCV-exposed and non-exposed adult *T*. *palmi*, larvae were given a 24-h acquisition access period (AAP) on CaCV-infected and non-infected *Chenopodium quinoa* leaves. Briefly, 5–6 weeks old *C*. *quinoa* plants were mechanically inoculated with a crude extract of CaCV-infected symptomatic *C*. *quinoa* leaves and kept in a growth cabinet at 25°C with 16 h/8 h light/dark photoperiod until symptom development. Cohorts of larvae (<12 h) were obtained from thrips that fed on healthy bean leaves. Batches of 100 larvae were transferred into Petri plates each containing a *C*. *quinoa* leaf placed on wet tissue paper. Larvae were given 24 h AAP on CaCV-infected leaves that developed chlorotic lesions seven days after inoculation. As control, larvae were allowed to feed on uninfected leaves. At least 1000 CaCV-exposed and non-exposed larvae were transferred to fresh bean seedlings contained in 2-L beakers and reared until adulthood in separate growth cabinets at 25°C with 70% relative humidity and 16 h/8 h day/night. Infection status of batches of virus-exposed and non-exposed thrips for the presence or absence of CaCV was determined by reverse transcription polymerase chain reaction (RT-PCR) using RNA extracted from sub-samples of each batch of thrips.

### Total RNA extraction and library preparation

Virus-exposed and non-exposed adult thrips were collected separately as batches of 100 individuals into 1.5 ml microfuge tubes to obtain three biological replicates for the two treatments. All samples were immediately processed independently. Total RNA was extracted using TRIzol reagent (Life Technologies) following manufacturer’s instructions. RNA extracts were treated with DNase using Turbo DNA-free kit (Ambion, Thermo Fisher Scientific) following manufacturer’s protocol. RNA was quantified using NanoDrop 3000 (Thermo Fisher Scientific). CaCV infection in all RNA samples was assessed using One-step RT-PCR kit (GeneMark) with CaCV-N gene-specific primers [CaCV-N-F1: ATGTCTAACGTCAGGCAACTT and CaCV-N-R1: CACTTCTATAGAAGTACTAGG [32]. Total RNA (2.5–3.0 μg) from three biological replicates of virus-exposed and non-exposed *T*. *palmi* was shipped on dry ice from Taiwan to the Australian Genome Research Facility (AGRF, Melbourne) for cDNA library preparation, Illumina sequencing, transcriptome assembly and expression profiling. Rest of the total RNA was stored at -80°C until quantitative PCR (qPCR) analysis.

### Illumina sequencing

Illumina cDNA libraries were prepared from total RNA by AGRF following the protocols for TruSeq RNA v2 (2014). Briefly, mRNA in total RNA preparations was enriched by using oligo dT beads prior to library preparation. Purified mRNA was then fragmented with a combination of divalent cations and heat and cDNA was synthesized. First strand cDNA was synthesized by random priming. Six cDNA libraries were prepared from poly(A) mRNA of three replicates each of virus-exposed and non-exposed adult thrips. The six libraries were multiplex-sequenced in one lane of an Illumina HiSeq 2000 sequencer to generate 100-bp paired-end reads using bclsfastq 2.17.1.14 pipeline. Quality control (QC) of resulting sequence reads was done according to AGRF QC standards, Phred 30 across all samples for 100 bp reads [33, 34]. High quality reads were further screened for the presence of any Illumina adapters/overrepresented sequences and CaCV sequences that were then removed.

### *De novo* assembly of *T*. *palmi* transcriptome

High quality reads from the six libraries were enriched as described below prior to *de novo* assembly of *T*. *palmi* reference transcriptome. Random errors in Illumina sequencing were corrected by Recorrector software using a k of 31 [35] followed by adapter trimming using Trimmomatic with Phred cut-off ≤ 2 [36]. Following enrichment, reads were *de novo* assembled using Trinity (v2.2.1), specifying the library type [37]. Quality of the *de novo* assembly was evaluated using TransRate by mapping all reads to the assembly which gave 0.46 optimal score with 0.38 optimal cut-off [38].

### Differential expression analysis

To determine differentially expressed (DE) transcripts in response to exposure to CaCV, reads from 6 Illumina libraries were individually mapped to the *de novo* assembled *T*. *palmi* reference transcriptome using TopHat (v2.0.14) software [39]. Number of Illumina reads that mapped to each contig of the reference transcriptome were estimated and counts were summarized at gene level across the three biological replicates using the featureConts (v1.4.6-p5) [40] utility of the Subread package [41]. Transcripts were assembled with the Stringtie tool v1.1.4 utilizing the reads alignment and in a *de novo* fashion [42].

DE transcripts between replicates of virus-exposed and non-exposed thrips were determined using Cufflinks tools [39]. Expression values were normalized as read counts per gene per sample with fragments per kilobase of exon per million mapped reads (FPKM). Significantly DE genes were identified using a binary statistical assessment. Briefly, a p-value was calculated for each gene in each sample and each comparison. Then p-values were corrected for multiple tests and comparisons (q-value) using false discovery rate (FDR). In this study, a FDR 0.05 cut off was used to determine p-value threshold. The correlation between virus-exposed and non-exposed transcripts was determined by calculating a Pearson correlation coefficient value [43].

### Provisional functional annotation of DE transcripts

Stringent filtering criteria were used to select highly significant DE transcripts for functional annotation. DE transcripts were selected by setting a cut off q-value < 0.01, log_2_-fold change (FC) > 1 and FPKM > 10, and were classified into functional categories using Blast2GO (B2GO) with default parameters [44]. Initially, transcripts were searched for sequence similarities in the NCBI non-redundant (nr) database using Blastx algorithm. Then transcripts were annotated by retrieving gene ontology (GO) terms associated with BLAST hits using GO databases in NCBI, nr reference protein database including PSD, UniProt, Swiss-Prot, TrEMBL, RefSeq, GenPept and DBXRef. Annotations were further improved by merging InterPro protein signatures and fine-tuned by using Annex-based GO term augmentation followed by removal of First Level GO terms. Enzyme codes were assigned for annotations using B2GO to identify which biological pathways are effected by DE enzymes and these were mapped to the Kyoto Encyclopaedia of Genes and Genomes (KEGG) database [45].

### Validation of RNA-Seq expression data with real-time RT-qPCR of selected transcripts

RNA-Seq expression levels of six randomly selected transcripts were validated using RT-qPCR. Actin, β-tubulin and 40S ribosomal protein S14 (RPS 14) were selected as non-DE genes from the dataset as internal references. Primers for target and reference genes were designed using Primer3 [46, 47]. Primer sequences are listed in S1 Table. Complementary DNA was synthesized using oligo dT primers and Superscript III First-strand cDNA synthesis kit (Life Technologies) using the same total RNA preparations (DNase-treated) used for Illumina sequencing. SensiFAST SYBR No-ROX Kit (Bioline) was used in a Rotor-Gene Q real-time PCR cycler (Qiagen) with 20 μl volumes containing 1 μl (10 ng) cDNA, 0.8 μl of each primer (10 μM), 7.4 μl of DNase- and RNase-free water and 10 μl of 2x SYBR No-ROX mix. Reaction conditions were 2 min at 95°C followed by 40 cycles of 95°C for 5s, 60°C for 10s and 72°C for 20s. Experimental design and subsequent data analysis methods were adopted from a previously described protocol [48]. Three biological replicates and two technical replicates were used per sample. Since initial experiments showed stable gene expression for all reference genes across all samples and treatments, actin was selected for future experiments. Real-time PCR amplification efficiencies of target genes and actin were determined by the standard curve method using a ten-fold dilution series of cDNA (from 50 ng to 0.001 ng). PCR efficiencies were calculated from the slopes of standard curves. Threshold cycle (Ct) number was determined from log scale amplification curves. Reaction efficiencies for target and reference genes showed 95–100% efficiency for 1–50 ng of cDNA template input. Hence, we used 10 ng of cDNA template for further experiments. For each reaction, no-template and no-RT control samples were included. Relative expression levels of target genes were calculated as 2^−(*Ct of target−Ct of reference*)^ [49]. Fold changes in gene expression between treatment and control were calculated using the 2^−ΔΔ*Ct*^ method; 2^−(Δ*Ct of treatment*−Δ*Ct of control*)^ [49]. For validation, qPCR derived log_2_-fold changes were compared with log_2_-fold values obtained by RNA-Seq analysis. Quantitative PCR results were compared with RNA-Seq data and Pearson correlation coefficient R and associated p values were calculated [43].

## Results

### *De novo* transcriptome assembly and differential gene expression

**Fig 1:**
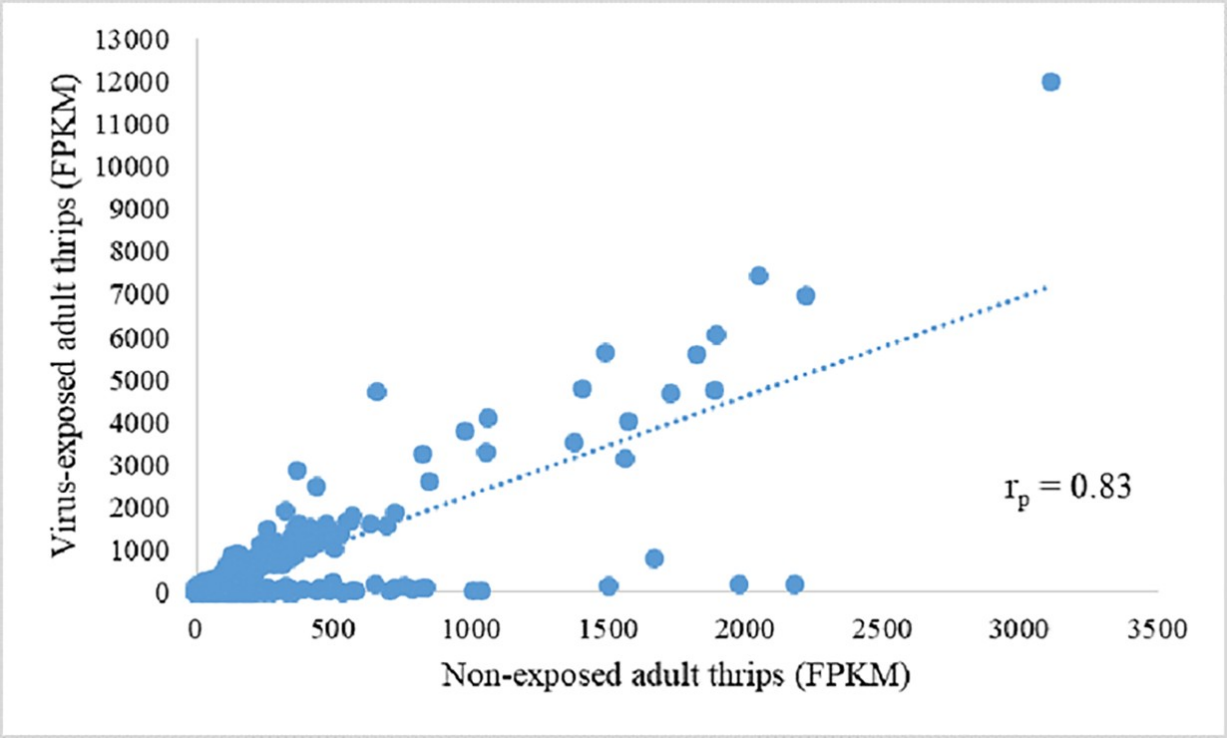
Comparison of normalized read counts (FPKM) between CaCV-exposed and non-exposed adults of *Thrips palmi*. Each point represents one transcript with log_2_ fold change > 1 in relative abundance between the two treatments and a q-value < 0.01. r_p_ = Pearson’s correlation coefficient.

*Thrips palmi* transcriptome was *de novo* assembled using 243,546,011 paired-end reads of 100 bp (48.71 Gb of sequence) from three biological replicates of cDNA libraries consisting of virus-exposed and non-exposed thrips. The raw sequence read data generated from the six samples have been deposited in the NCBI Sequence Read Archive as BioProject PRJNA498538 with accession numbers SAMN10316162—SAMN10316167. Per base sequence quality of all six libraries was >93% bases above the Phred quality score of 30. Assembly of high quality reads generated 166,445 contigs with an average length of 918 bp and an N50 of 2114 bp (Table 1). Cleaned reads from individual libraries were aligned to the *de novo* assembled contigs to estimate read counts that mapped to contigs. All six libraries aligned to the reference transcriptome with at least 75% concordant pair alignment rate. Quality assessment of Cufflinks data showed fairly good quality in all the six libraries. Collectively, there was a positive correlation (Pearson correlation, r_p_ = 0.83, *P* < 0.0001) between the normalized read counts (FPKM) of the virus-exposed and non-exposed treatments for transcripts that exhibited greater than 2-fold change in expression with a q-value < 0.01, and the virus treatment tended to have a larger range of FPKM values, indicating significant perturbation (Fig 1). For stringency, a transcript was considered DE when the log_2_-fold change was >1.0, the q-value was < 0.01, and FPKM >10 for at least one of the treatments (CaCV-exposed or non-exposed) in the pairwise comparison. With these criteria, we identified 1,389 DE transcripts, of which 708 were up-regulated and 681 were down-regulated in virus-exposed thrips.

**Table 1:** Summary statistics for *T*. *palmi de-novo* assembled transcriptome.

Assembly feature	Statistic
Total assembled contigs	166,445
Total assembled bases	152,899,637
Mean contig length	919 bp
N 50 contig length	2,114 bp
No. contigs with predicted ORFs	39,449
No. contigs ≥ 400 bp with predicted ORFs	32,262
Blastx matches (*E* ≤ 10^−5^)	22,582
B2GO annotations	10,407

### Annotation of DE transcripts

**Fig 2:**
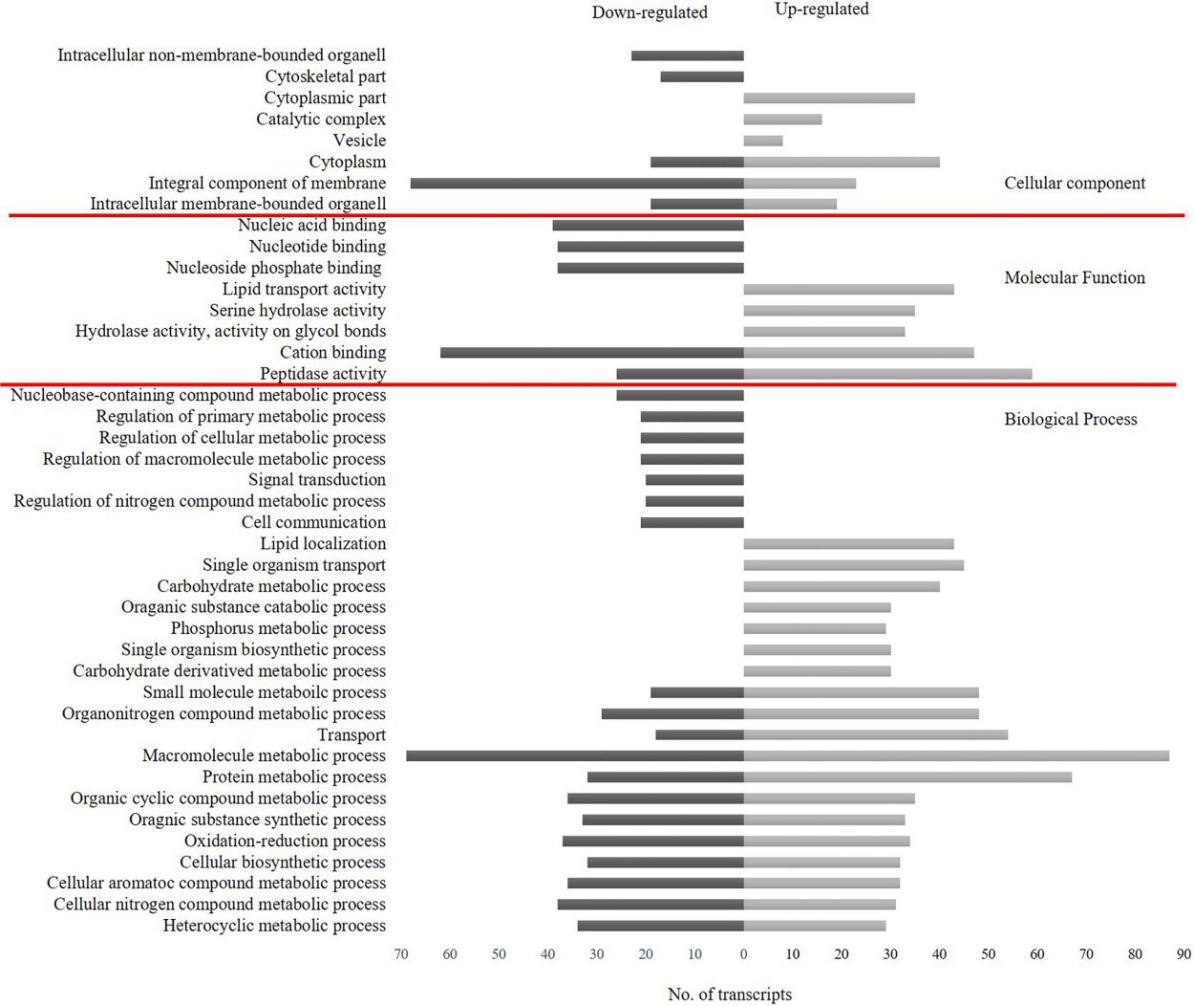
Number and functional categories of gene ontology terms assigned for up-regulated and down-regulated transcripts of CaCV-exposed *Thips palmi* at level 2.

Of the 1,389 identified DE transcripts, 430 (60.7%) up-regulated and 385 (56.5%) down-regulated transcripts matched sequences in the NCBI non-redundant sequence database. Among the DE transcripts that had Blastx hits, 316 up-regulated and 287 down-regulated transcripts were annotated. Forty-five GO terms categorized into biological process (BP), molecular function (MF) and cellular component (CC) were assigned for DE transcripts at level 2. The most dominant GO terms were ‘metabolic process’ in BP domain, ‘peptidase activity’ in MF domain and ‘cytoplasm’ and ‘cytoplasm part’ in CC domain (Fig 2). Within the BP category, GO terms associated with regulation of metabolic processes, signal transduction and cell communication were all down-regulated, whereas lipid localization, carbohydrate/derivative metabolic process, phosphorous metabolic process, single organism transport and biosynthetic process were all up-regulated. Other transcripts that fell into GO terms ‘transport’, ‘metabolic processes’, ‘cellular biosynthetic process’ and ‘oxidation-reduction’ had both up- and down-regulated transcripts. In the MF category, transcripts assigned with GO terms ‘nucleic acid/nucleotide binding’ and ‘nucleoside phosphate binding’ were all down-regulated, whereas ‘lipid transport activity’ and ‘hydrolase activity’ were all up-regulated. In the CC category, GO terms such as ‘integral component of membrane’, ‘cytoskeletal part’ and ‘intracellular non-membrane-bounded organelle’ were among the down-regulated GO terms and cytoplasm and cytoplasmic part were among the up-regulated GO terms. Mapping enzymes into KEGG pathways [45] revealed the most enriched pathways among the up-regulated transcripts were ‘biosynthesis of antibiotics’ and ‘purine metabolism’ with 10 enzymes placed in each. Of the down-regulated transcripts the most enriched pathways were ‘purine metabolism’, ‘tyrosine metabolism’ and ‘isoquinoline alkaloid biosynthesis’ with 3 enzymes placed in each.

Following GO term assignment, 186 up-regulated DE transcripts could be categorized into three groups that may infer roles in innate immunity, salivary gland processes, and thrips fitness and fecundity based on Blastx descriptions and GO terms (Table 2). The majority of the most highly down-regulated transcripts had Blastx annotations for structural constituents of the cuticle (Table 3). In addition, there were chitinases and nucleic acid binding genes among the down-regulated transcripts.

**Table 2:** List of significantly up-regulated DE (q < 0.01, log_2_-FC > 1.0, FPKM >10) transcripts categorized based on Blastx descriptions and GO terms that are predicted to be associated with innate immunity, salivary glands and fitness and fecundity in CaCV-exposed and non-exposed *Thrips palmi* transcriptome.

Putative pathway	Blastx description (no. of transcripts)	FPKM (non-virus-exposed)	FPKM (virus-exposed)	log_2_- FC	GO term	Putative function
**Cellular immunity**						
**Lysosome**	Cathepsin B (10)	40.9–501.4	166–1188.8	1.0–2.0	Viral entry into host cells, Proteolysis, Endopeptidase activity, Lysosome	Innate immunity [50]
	Lipase 1 (4)	3.2–33.6	20.7–82.5	1.3–2.7	Hydrolase activity, Lipid metabolic process, Membrane	Antiviral [51], innate immunity [52]
	Lysosomal aspartic protease (3)	316.5–360.9	934.4–1127.5	1.6	Proteolysis, Lysosome	Innate immunity [53]
	Lysozyme C (2)	37.2–341.7	115–784.3	1.2	Lysozyme activity	Innate immunity [54–56]
	Acid-phosphatase-1	8.4	20.8	1.3	Acid phosphatase activity	Antimicrobial [57]
	Beta mannosidase	4.3	10.6	1.3	CHO metabolic process	Innate immunity [58]
**Melanisation**	Phenoloxidase 2 (6)	2.3–42.5	10.0–91.0	1.0–2.3	Melatonin encapsulation of foreign target, L-DOPA monooxygenase activity, Defence, Oxidation-reduction	Innate immunity [59]
	Phenoloxidase subunit A3	16.4	51.8	1.7	Oxidation-reduction	Innate immunity [59]
	Serine protease ester-like	14.9	47.8	1.7	Proteolysis, Melanisation defence response	Innate immunity [60], Salivary gland [61]
	Glucose dehydrogenase [quinone] (9)	5.2–77.8	12.8–193.4	1.0–1.6	Oxidation-reduction process	Innate immunity [62]
	Poly(U)-specific endoribonuclease homologue (3)	93–11.0	31.7–38.2	1.8	Hydrolase activity	Innate immunity [63]
	Laccase-5 isoform X1	19.4	61.5	1.7	Oxidation-reduction process	Innate immunity [64, 65]
	Troponin isoform 1 like (2)	13.7–65.8	28.4–208.1	1.0–1.6	Protein binding, Calcium binding	Innate immunity [66]
	Serine protease 13	143.0	354.3	1.3	Proteolysis, Serine-type peptidase activity	Innate immunity [67]
**Humoral immunity**						
**Complement and coagulation cascades**	Carboxypeptidase B like (3)	4.5–146.3	27.8–328.0	1.3–3.3	Proteolysis, Metallo-carboxypeptidase	Innate immunity [68]
	Coagulation factor ix	11.6	31.0	1.4	Zymogen activation, Extracellular exosome	Innate immunity [69]
	Limulus clotting factor C like	11.1	31.6	1.5	Protein binding	Innate immunity [70]
**Immune system pathway**						
**Toll**	Defensin 2	10.4	47.2	2.2	Defence response	Innate immunity [65]
	Gram-negative bacteria-binding 3-like	132.8	469.8	1.8	CHO binding	Innate immunity [71]
	Serine protease 44	5.9	18.9	1.7	Proteolysis, Serine-type peptidase activity	Innate immunity [72, 73]
**Antigen processing and presentation**	70 kDa heat shock partial	20.2	54.7	1.4	ATP binding	Antiviral [74]
	Heat shock 70 a1 partial	54.7	113.0	1.0	ATP binding, Extracellular exosome	Antiviral [74]
	Heat shock cognate 71	31.7	90.6	1.1	ATP binding	Antiviral [55]
**Other innate immunity-related genes**						
**Serine proteases**	Serine protease (4)	15.3–95.8	31.4–218.7	1.0–1.7	Proteolysis, Serine-type peptidase activity	Innate immunity [75]
	Serine protease 12 -like (3)	4.2–37.8	13.0–109.9	1.6	Proteolysis, Serine-type peptidase activity	Innate immunity [76]
	Serine protease 9-like (5)	8.2–38.6	21.0–92.9	1.3–1.5	Proteolysis, Serine-type peptidase activity	Innate immunity [76]
**Trypsins**	Trypsin-like serine protease (3)	2.6–26.2	12.8–73.5	1.5–2.3	Proteolysis, Serine-type peptidase activity	Innate immunity [77]
	Trypsin	7.6–32.0	16.1–85.0	1.0–1.5	Proteolysis, Serine-type peptidase activity	Innate immunity [78]
Apolipo D-like (2)		12.8–29.1	43.6–78.3	1.4–1.8	Pigment binding	Innate immunity [56]
Chitin binding peritrophin-A domain containing (2)		33.0–48.3	110.0–211.8	1.7–2.1	Chitin metabolic process	Innate immunity [79, 80]
Chymotrypsin protease (4)		8.7–28.5	22.1–89.1	1.3–1.6	Proteolysis	Innate immunity [81]
Cytochrome P450 4C1 (2)		3.5–10.9	15.7–31.7	1.5–2.1	Oxidation-reduction process	Immunity [82]
Cytochrome P450 6k1-like		313.5	634.1	1.0	Oxidation-reduction process	Immunity [82]
Extracellular serine threonine kinase FAM20C		44.2	135.8	1.6	Protein phosphorylation	Immunity [83]
Esterase FE4-like		6.0	16.7	1.5	Hydrolase activity	Innate immunity [80]
Facilitated trehalose transporter Tret1-like		9.7	19.6	1.0	Transmembrane transport	Immunity [84]
Elicitin 6 partial		12.2	33.0	1.4	Defence response, Chitin binding	Defence response in plants [85]
Pathogenesis-related protein 5-like		5.8	15.8	1.4	Systemic acquired resistance, Response to virus, apoplastic	Defence response in plants [86]
**Salivary gland-associated**						
Lipase 3 (15)		2.5–71.8	12.3–347.4	1.0–2.3	Lipid metabolic process, Hydrolase activity	General digestion [87, 88]
Seine protease (9)		5.9–95.8	18.8–218.2	1.0–1.7	proteolysis	
Trypsin-like serine protease (3)		2.6–26.2	12.8–73.4	1.5–2.3	Proteolysis, Serine-type peptidase activity	
Carboxypeptidase (3)		4.5–146.4	27.8–328.0	1.3–3.3	Proteolysis	
Pectin lyase (4)		32.4–216.8	68.1–507.0	1.0–1.5	Polysaccharide catabolic process	Digestion of plant cell wall [88]
β-glucosidase		2.3	12.0	2.3	Carbohydrate metabolic process	
Endoglucanase (4)		66.5–152.1	166.7–339.2	1.2–1.3	Fructose, Sucrose metabolic process, Cellulase activity	
α-amylase A-like		69.0	186.8	1.4	Sucrose metabolic process, Extracellular exosome	Sugar metabolism [88]
Angiotensin-converting enzyme-like		8.4	17.0	1.0	Proteolysis	Detoxification and inhibition of plant defence [89]
Phosphoribosyl formylglycinamidine synthase		24.7	56.2	1.2	*'de novo'* IMP biosynthetic process	Advantageous for infecting virus [90]
Uridine phosphorylase 1 isoform X1		3.1	10.9	1.8	Nucleoside metabolic process	Salivary gland [91]
Pyridoxal phosphate phosphatase		8.3	46.7	2.5	Dephosphorylation, Hydrolase activity	Salivary gland expressed gene [92]
**Fitness and fecundity-related genes**						
Vitellogenin (54)		3.1–2446.1	11–7446	1.0–2.6	Lipid transport	Reproduction [93,94], Innate immunity [95]
**Other genes**						
LPXTG-domain-containing cell wall anchor partial		28.0	77.7	1.5	Phosphorylation	Cell surface adhesion [96]

FC = fold change of virus-exposed treatment relative to non-virus control treatment.

**Table 3:** List of significantly down-regulated DE (q < 0.01, log_2_-FC > 1.0, FPKM >10) transcripts in CaCV-exposed and non-exposed *Thrips palmi* transcriptome.

Blastx description (no. of transcripts)	FPKM (non-virus-exposed)	FPKM (virus-exposed)	log_2_- FC	GO term
Larval cuticle A2B-like (3)	117.1–220.8	0.3–1.8	9.3–8.0	Structural constituent of cuticle
Serine protease inhibitor 3 4 isoform X1	44.2	0.2	7.9	Extracellular space
Pupal cuticle C1B-like (2)	29.4–100.6	0.1–0.6	7.4–7.6	Structural constituent of cuticle
Endocuticle structural glyco bd-8-like	53.6	0.4	7.1	Structural constituent of cuticle
Alpha-tocopherol transfer -like	28.2	0.2	7.0	Transport
Collagen alpha-1(III) chain-like	47.2	0.5	6.5	Chitin binding, Extracellular region
Uncharacterized protein LOC106678716	29.6	0.3	6.4	Integral component of membrane
Apolipo D-like	85.8	1.0	6.4	Pigment binding
Endocuticle structural glyco bd-4-like (2)	29.8–203	0.4–2.8	6.2–6.3	Structural constituent of cuticle
Uncharacterized protein LOC103510819	24.2	0.3	6.1	Integral component of membrane
Trypsin	24.2	0.4	6.0	Proteolysis; serine-type endopeptidase activity
Serine ase stubble	72.8	1.1	6.0	GTPase activity, Proteolysis, Serine-type endopeptidase activity
Probable chitinase- 3	19.4	0.3	6.0	Chitinase activity, Extracellular region
Fibroin heavy chain	32.7	0.5	5.9	Structural constituent of cuticle
Cuticle 7	49.6	0.8	5.9	Structural constituent of cuticle
Glycine-rich cell wall structural -like	37.4	0.7	5.8	Anatomical structure development
Cuticular analogous to peritrophins 1-G	12.5	0.2	5.7	Chitin binding, Chitin metabolic process, Extracellular region
Cytochrome P450 4g15	26.1	0.5	5.7	Iron ion binding, Oxidation-reduction process
Serine ase stubble isoform X2	16.7	0.4	5.5	GTP binding, Serine-type endopeptidase activity
Location of vulva defective 1 isoform X2	12.7	0.3	5.5	Membrane, Integral component of membrane
COPII coat assembly partial	87.2	1.9	5.5	Neuropeptide signaling pathway
Sal 1	20.9	0.5	5.5	Nucleic acid binding
Glucose dehydrogenase [quinone]-like	14.5	0.3	5.4	Oxidation-reduction process
Proclotting enzyme-like	14.8	0.3	5.4	Proteolysis, Serine-type endopeptidase activity
Larval cuticle A3A-like	24.2	0.7	5.2	Structural constituent of cuticle
Glycine-rich cell wall structural -like	12.9	0.3	5.2	Chitin-based cuticle development, Structural constituent of chitin-based cuticle
Endocuticle structural glyco bd- partial	168.6	4.8	5.1	Structural constituent of cuticle, Extracellular space
Osiris 14	15.2	0.4	5.1	Integral component of membrane
Cuticular precursor	24.1	0.7	5.1	Structural constituent of cuticle, Nucleic acid binding
Endocuticle structural glyco bd-2-like	33.4	1.0	5.0	Structural constituent of cuticle, Extracellular space

FC = fold change of virus-exposed treatment relative to non-virus control treatment.

### Real-time quantitative PCR

**Fig 3:**
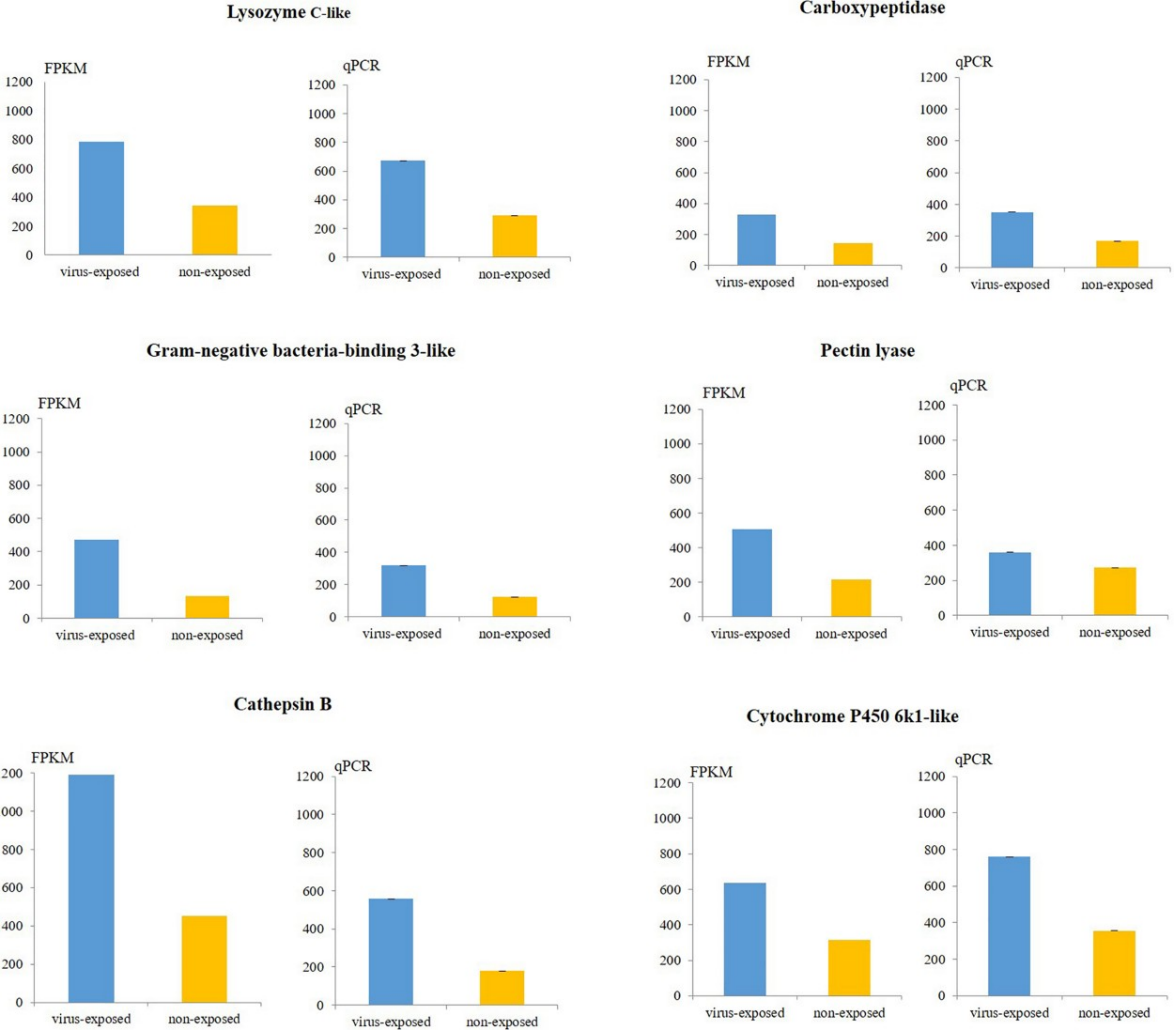
Validation of RNA-Seq gene expression by qPCR. FPKM values obtained by RNA-Seq analysis and relative expression levels obtained by qPCR for six selected genes in CaCV-exposed and non-exposed *Thrips palmi* are shown. Values were multiplied by a factor of 1000. Error bars represent the standard error for three biological replicates.

Fold changes of six randomly selected DE transcripts among the annotated genes were validated by real-time qPCR using the same total RNA preparations used for Illumina library preparation. Expression of target genes was normalized to actin internal reference gene selected from non-DE genes in the dataset. Relative expression levels and fold changes in target gene expression in virus-exposed and non-exposed thrips showed similar trends in qPCR and RNA-Seq for most transcripts tested. The exception was pectin lyase transcripts that showed a reduced ratio between virus-exposed and non-exposed expression levels in qPCR (Fig 3). Pearson correlation coefficient r_p_ was 0.658 (*P* = 0.003) indicating a significant positive correlation between the RNA-Seq and qPCR data.

### Comparison of DE transcripts between *T*. *palmi*-CaCV and *F*. *occidentalis*-TSWV interactions

A comparison of the adult transcriptomes of *T*. *palmi—*CaCV (this study) and *F*. *occidentalis*—TSWV [30] using tblastx revealed a small number of transcripts that were differentially up-regulated in both thrips—tospovirus interactions (Table 4). This suggests potentially conserved responses by different thrips species across genera against tospoviruses from different serogroups that may be suitable targets for novel generic pest control.

**Table 4:** List of conserved up-regulated transcript sequences in two adult-stage thrips—tospovirus interactions.

Tp transcript annotation	Focc—TSWV transcript code	Tp—CaCV transcript code	Log_2_ FC Focc	Log_2_ FC Tp	% sequence identity	Sequence coverage
DNase I	FOCC007280-RA	TP.8153.1	+ 2.62	+ 1.31	86	1186 nt
Uncharacterized protein LOC106129042	TCONS_00032732	TP.1784.1	+ 2.06	+ 2.47	81	194 aa
Endoglucanase	FOCC015899-RA	TP.19931.1, TP21769.1, TP.21770.1, TP.21771.1	+ 1.95	+ 1.2 to + 1.3	80	115 aa
Carbonic anhydrase	CUFF.8568.2	TP.33694.1	+ 2.88	+ 2.08	80	92 aa
Uncharacterized protein LOC105690123	CUFF.8228.1 CUFF.2322.1	>10 Isoforms >10 Isoforms	+ 2.58 +2.64	up up	62 60	< 200 aa < 200 aa
Hexamerin	FOCC009367-RA	TP.20198.1#	+ 4.20	+ 1.81	45	33 aa
Hemocyanin subunit type 1 precursor	FOCC002013-RA	TP.20202.1#	+ 13.31	+ 1.75	38	50 aa
Hexamerin, arylphorin subunit alpha	FOCC012829-RA	TP.25095.1#	+ 8.87	+ 1.1	38	45 aa

Focc = *Frankliniella occidentalis*; TSWV = tomato spotted wilt virus; Tp = *Thrips palmi*; CaCV = capsicum chlorosis virus; nt = nucleotides; aa = amino acids. FC = fold change of virus-exposed treatment in relative to non-virus control treatment.

# Annotated as phenol oxidase 2-like

The up-regulated genes common to both virus-vector interactions are associated with innate immunity and salivary glands. Hexamerins [log_2_ fold change 1.81 (*T*. *palmi*) vs 4.2 (*F*. *occidentalis*)] are multi-subunit storage proteins with phenol oxidase activity that are involved in insect innate immunity, humoral immune response to pathogens and disease resistance [59]. Similarly, hemocyanin subunit 1 precursor [log_2_ fold change 1.75 (*T*. *palmi*) vs 13.31 (*F*. *occidentalis*)] was shown to have phenol oxidase activity in melanogenesis [97]. Carbonic anhydrase [log_2_ fold change 2.08 (*T*. *palmi*) vs 2.88 (*F*. *occidentalis*)] has a physiological role in pH and ion regulation pathways. Endoglucanase [log_2_ fold change 1.2–1.3 (*T*. *palmi*) vs 1.95 (*F*. *occidentalis*)] is a salivary gland-associated cell wall digestive enzyme [88]. Four isoforms of this gene were identified in *T*. *palmi* transcriptome. Deoxyribonuclease I [log_2_ fold change 1.31 (*T*. *palmi*) vs 2.62 (*F*. *occidentalis*)] is an endonuclease that cleaves DNA and has been suggested to play a role in apoptosis [98]. This gene is also listed among the DE contigs with a log_2_ fold change of 2.82 associated with vector response to TSWV infection in *F*. *fusca* adult transcriptome [31], further validating the conserved response of up-regulating this thrips gene.

When down-regulated genes in adult transcriptomes were compared, none of the most highly down-regulated TP transcripts were shared between the two systems. However, genes of endocuticle structural glycoprotein [log_2_ fold change 5.0–7.1 (*T*. *palmi*) vs 4.5 (*F*. *occidentalis*)], which is a structural constituent of cuticle and alpha-tocopherol transfer-like [log_2_ fold change 7.0 (*T*. *palmi*) vs 4.9 (*F*. *occidentalis*)] which has been shown to be involved in antiviral immunity in *Drosophila melanogaster* [99] were down-regulated in *F*. *occidentalis* larvae [30] and *T*. *palmi* adults.

## Discussion

Quantifying changes in global gene expression is one means of inferring cellular and physiological responses of insects to infection by insect-pathogenic viruses or insect-vectored plant-viruses [100]. This is especially relevant for viruses like tospoviruses that circulate and propagate inside insect cells and tissue systems, exploiting host cellular machinery to replicate and to complete their life-cycles [101, 102]. This study presents the first transcriptome for an insect in the genus *Thrips*. We analysed *T*. *palmi* transcriptome in response to CaCV infection to identify DE transcripts and to classify these genes by sequence homologies (ontologies) to known proteins to begin to dissect the global response of this thrips species to tospovirus infection. Here, we present hypotheses about the effect of virus infection on three dynamic processes in vector biology: innate immunity, growth and development, and fitness.

### Innate immunity-related genes

Based on Blastx descriptions and GO terms for DE transcripts, we identified 86 up-regulated transcripts putatively associated with innate immune response that may have been triggered by CaCV infection (Table 2). The majority of these genes are likely involved in cellular immunity activated in lysosomes such as cathepsin B [50, 103, 104], lysosomal aspartic proteases and lysozyme C. We identified 10 cathepsin B genes that were up-regulated in virus-exposed *T*. *palmi*. Cathepsins are known to be involved in various biological processes including protein degradation, apoptosis and signalling activated in late endosome and lysosome and implicated in virus entry and replication [105, 106]. Therefore, it appears that CaCV infection may lead to cytopathological effect in thrips cells through cell damage and apoptosis. Another set of genes that were up-regulated in virus-exposed thrips encode glucose dehydrogenases that are known to participate in the initiation of cellular immune responses by encapsulation of pathogens [62].

Genes associated with melanisation seem to play a significant role in virus-exposed *T*. *palmi*. Besides pigmentation, melanin deposits and encapsulates foreign targets such as nematodes, parasitoids and virus-infected tissues and inhibits progression of infection [59, 107]. Enzymes such as phenoloxidases (PO) [59], serine proteases (SP) [60] and laccase [64] are the key players in the melanisation pathway. PO generate highly cytotoxic quinones that can inactivate invading viral pathogens [108]. In *T*. *palmi* transcriptome, we identified several PO genes and a CLIP domain-containing SP that were up-regulated in virus-exposed thrips. Previous studies have shown that PO cascade is activated in mosquitoes in response to Semliki Forest virus infection resulting in inhibition of virus spread in cell culture [109, 110]. CLIP domain-containing SP induce melanisation immune response by activating PO [59, 60]. In addition to PO and SP, other genes that participate in the melanisation process were identified, such as poly(U)-specific endoribonuclease homologues and laccase-5 [63–65].

Genes involved in other innate immune system pathways such as Toll, antigen processing and presentation, and complement and coagulation cascades (humoral immunity) were also found up-regulated. Among the up-regulated transcripts in CaCV-exposed thrips, 63 encoded proteolytic enzymes, including 30 serine protease and trypsin genes. According to GO assignment all serine proteases and trypsins in this thrips transcriptome were predicted to exhibit serine-type endopeptidase activity. The role that these proteases may play in virus-exposed thrips is not known. Previously it was shown that insect serine proteases and trypsin-like serine proteases are generally involved in hemolymph coagulation, activation of antimicrobial peptide synthesis, and melanin synthesis [75, 111, 112]. Therefore, these genes may have roles in thrips humoral immunity. Interestingly, among the immune-related genes, two genes showed >67% similarity to plant genes encoding pathogenesis-related (PR) protein 5 and elicitin 6.

Our study of *T*. *palmi* identified activation of different immunity-related pathways than the results reported by Zhang and collaborators [52] for *F*. *occidentalis-*TSWV, such as Toll, JAK-STAT and RNA interference. Either these pathways have no significant role in adult *T*. *palmi* against CaCV or they were active only in larvae at the time of virus acquisition and initial replication and intercellular movement, which was not captured by our adult transcriptome. In another study, Schneweis and colleagues [30] showed that in adult *F*. *occidentalis* exposed to TSWV, 75% of innate immune response related transcripts were annotated as insect storage proteins, hexamerins which are also associated with humoral immunity. We did not identify hexamerins among the DE transcripts of *T*. *palmi* suggestive of activation of different set of genes in *T*. *palmi*’s innate immunity. When compared with *F*. *fusca-*TSWV adult DE genes, we identified up-regulation of several similar immune system-related genes such as cathepsin B, aminopeptidase N, serine proteases and heat shock protein 70. Therefore, it is likely that more generally in adult thrips infected with tospoviruses, immune reactions like apoptosis, phagocytosis and proteolysis are activated.

### Salivary gland-associated genes

Salivation is an essential process in insect feeding [113, 114] and virus inoculation of hosts [28, 115]. Various components of saliva are involved in extra-oral digestion of plant tissues and suppression or detoxification of host defence responses [116, 117]. In the present study, the majority of up-regulated putative salivary gland genes identified in this study appear to be involved in digestion (Table 2). Of the 20 lipases, up-regulated in virus-exposed thrips, 15 were lipase 3-like, 4 transcripts corresponded to lipase 1, and 1 was a H-B-like lipase. Lipases are also known to be involved in innate immunity [51]. To identify lipases that may be secreted components of saliva, signal peptide sequences were predicted *in silico*. All lipase 1 sequences lacked a signal peptide, hence were considered unlikely to be components of saliva and more likely involved in innate immunity. Of the 15 lipase 3 genes, nine sequences contained a signal peptide and a predicted cleavage site at their N-terminus, suggestive of secreted proteins. Lipases have previously been identified to be associated with salivary glands of other insects, including the potato leafhopper (*Empoasca fabae*) [87], whitefly (*Bemisia tabaci*) [61] and *F*. *occidentalis* [88]. Other large groups of enzymes that may be involved in general digestion are proteolytic enzymes, serine proteases and carboxypeptidases. Eleven of 15 serine proteases and trypsin-like serine proteases and carboxypeptidases in virus-exposed *T*. *palmi*, contained predicted signal peptides and cleavage sites. Similar proteins were identified in salivary gland transcriptomes of *E*. *fabae* and *F*. *occidentalis* [87, 88]. One of the serine proteases contained a CLIP domain. Because CLIP domain-containing serine proteases are known to induce melanisation [59] and the presence of a predicted signal peptide cleavage site, this protease may be a component of saliva which provides immunity by activation of Toll signalling pathway or PO cascade leading to melanisation [118, 119]. Up-regulation of several transcripts encoding secreted lipases and proteolytic enzymes implies that they participate in general digestion of lipids and proteins during thrips feeding.

Plant-feeding insects secrete a range of cell wall-degrading enzymes to release cell contents and to facilitate subsequent ingestion [120]. Pectin is one of the major polysaccharides in the plant cell wall and middle lamella [121]. Pectin lyase is a pectin-hydrolysing enzyme expressed in salivary glands of phytophagous insects, including *F*. *occidentalis* [88], *E*. *fabae* [87] and the plant bug *Lygus hesperus* [122]. Other cell wall degrading enzymes investigated from insects are laccase, β-glucosidase and endo-β-glucanase [87, 88, 123–125]. Presence of transcripts encoding pectin lyases, β-glucosidases and endoglucanases in the *T*. *palmi* transcriptome is consistent with findings in other insects. Based on signal peptide predictions, some of these enzymes may be secreted, a requisite for extra-oral digestion.

A α-amylase that may be involved in sugar metabolism was identified among up-regulated transcripts in virus-exposed thrips. This sequence lacked an obvious signal peptide, but its GO term ‘extracellular exosome’ suggests extracellular localization and a putative digestive role. α-amylases have been identified in the saliva of many insects including honeybee (*Apis melifera*) [126], mosquito (*Ades aegypti)* [127], silkworm (*Bombyx mori*) [128] and red flour beetle (*Tribolium castaneum*) [129], and also in salivary gland transcriptomes of thrips *F*. *occidentalis* [88] and *E*. *fabae*, [87]. Genes encoding several other sugar metabolic enzymes such as maltase, sucrase and β-glucosidase were identified in the *F*. *occidentalis* salivary gland transcriptome [88] but were not detected among the significantly DE genes in the *T*. *palmi* transcriptome.

Among the other salivary gland-associated genes identified in this study were angiotensin-converting enzyme-like (ACE) genes which may have a role in inhibition of plant defence [89, 91]. Wang and colleagues [89] suggest that pea aphid secreted ACE may digest plant peptide hormones in phloem sap and function as signal molecules to mount defence response against herbivorous insects, thereby supressing plant immune responses. The *T*. *palmi* ACE may have similar functions.

### Fitness and fecundity-associated genes

Among the up-regulated genes in CaCV-exposed thrips, 54 transcripts represented vitellogenin (Vg) homologues (Table 2). Vg contigs were also highly up-regulated (log_2_ fold change >9) in adult and pupal transcriptome of *F*. *fusca* infected with TSWV [31]. The primary role of insect Vg is to transport egg yolk protein components synthesized extra-ovarially in fat bodies into growing oocytes by receptor-mediated endocytosis [130]. In female insects Vg is one of the most abundant proteins [131]. Accumulation of Vg in vesicles in insects is mediated by receptors of the low-density lipoprotein receptor (LDLR) family [111]. Among the up-regulated genes in virus-exposed thrips we identified two Vg receptors and a LDLR; one of the Vg receptor genes was highly abundant with a FPKM value of 1598 and a log_2_-fold change of 2.0. Abundance of Vg transcripts and their receptors in virus-exposed *T*. *palmi* implies transovarial transport of egg yolk protein components to the growing oocytes had been promoted in the presence of CaCV infection. Enhanced vitellin and vitellogenin levels in response to a plant virus infection was first reported from whitefly, MEAM1 (B biotype) *B*. *tabaci* that fed on tomato yellow leaf curl China virus-infected plants [93]. These authors speculated that the vector benefits from virus infection due to increased longevity and fecundity. A recent study has shown that TSWV has positive effects on the fecundity of *F*. *occidentalis* [132]. In contrast, leafhopper, *Recilia dorsalis* that fed on rice gall dwarf virus (RGDV)-infected plants showed significantly reduced longevity and fecundity associated with reduced levels of Vg compared to non-viruliferous individuals [94].

There are other reports that have shown involvement of Vg in immune responses of insects including citrus whitefly (*Dialeurodes citri*) [50], *A*. *aegypti* [95] and honeybee (*Apis mellifera*) [133]. Based on these precedents, enhanced Vg gene expression in virus-exposed *T*. *palmi* may play a role in innate immunity to CaCV infection. In support, Vg was found to be 9-fold up-regulated in pupae of *F*. *fusca* exposed to TSWV [31]. Another known role that Vg plays in plant virus-vector interactions is facilitation of vertical virus transmission, exemplified by transovarial transmission of rice stripe virus in small brown planthopper (*Laodelphax striatellus*) [134]. Similar to our findings in virus-exposed *T*. *palmi*, Vg was the most abundant transcript in virus-exposed small brown planthopper transcriptome [135]. Previously, it has been shown that TSWV is not vertically transmitted from viruliferous adult females to eggs [22]. Further, there is no evidence for vertical transmission in other tospovirus-thrips pathosystems and it is generally accepted that tospoviruses are not transovarially transmitted. However, existence of transovarial transmission of CaCV in *T*. *palmi* cannot be excluded due to a current lack of knowledge in tissue tropism and virus localization in the body of *T*. *palmi*. Furthermore, Vg was not among the most abundant unigenes and DE genes in *F*. *occidentalis* transcriptome infected with TSWV [52]. Therefore, it is possible that enhanced Vg expression is unique to CaCV-*T*. *palmi* interaction, but the exact role that Vg may play needs to be investigated further.

In summary, we have assembled a whole-body transcriptome of adult *T*. *palmi* and estimated differential abundance of transcripts when exposed to CaCV infection. The majority of DE transcripts did not contain Blastx annotations or conserved functional domains, but their differential abundance reflects unique roles in *T*. *palmi*-virus interaction and *T*. *palmi* biology in general. Although we cannot directly compare our data with studies of *F*. *occidentalis* [30] and *F*. *fusca* [31] exposed to TSWV, during the adult stages of these species, there were a few common GO terms assigned to DE transcripts such as hydrolase activity, nucleotide/nucleic acid binding, carbohydrate metabolic process and peptidase activity. In addition, the majority of annotations assigned to DE transcripts of *T*. *palmi* appeared to be species-specific. Aside from the inherent differences across thrips species and tospoviruses species, differences in the experimental methods and computational protocols and parameters used in data analyses could explain the weak overlap in transcripts responsive to virus across the three studies. For example, DE analyses for *F*. *fusca* [31] and *T*. *palmi* were performed using *de novo* transcriptome assemblies, whereas Schneweis and colleagues [30] used a draft genome reference for DE analysis for *F*. *occidentalis*.

We identified virus-responsive up-regulated DE transcripts that have putative roles in thrips innate immunity, feeding and fecundity. Down-regulated transcripts that are likely associated with structural component of cuticle and integral components of membrane may be responsible for delaying thrips development and increased longevity to ensure virus persistence and transmission. However, functions of those genes need to be experimentally validated in future. For example, transcripts associated with thrips feeding and fecundity could be candidates for potential targets in thrips and tospovirus management. In addition, transcriptomic data generated in this study will enrich genomic information of thrips and will allow functional studies on other economically important thrips and insects more broadly.

## Supporting information

S1 Table Sequences of oligonucleotide primers used in qPCR. (DOCX)
