# Supplementary material for: Transcriptome-wide responses of adult melon thrips (Thrips palmi) associated with capsicum chlorosis virus infection
Source: PLoS One. 2018 Dec 7;13(12):e0208538. doi: 10.1371/journal.pone.0208538 (PMC6286046; doi:10.1371/journal.pone.0208538)
Supplement: S1 Table — (DOCX) [file pone.0208538.s001.docx]

S1 Table Sequences of oligonucleotide primers used in qPCR

| **Gene** | **Forward (5’ to 3’)** | **Reverse (5’ to 3’)** | | **Amplicon size (bp)** | |
| --- | --- | --- | --- | --- | --- |
| Actin | AGTTGCCCCTGAGGAACAC | | CAGAGGCATACAGGGAAAGG | 152 | |
| β-tubulin | CCAGCCACATTCCTGGATAC | | ATGCGTTGGCAGTCACATAC | 117 | |
| 40S ribosomal S14 | GGAAGGTTCAGCAAGAGCAG | | TGTCGTTGAAACTGGCGTAG | 102 | |
| Lysozyme C-like | CAGACGGCTCCAAGGACTAC | | CCCACGTGTTCTTGCTCTTC | 180 | |
| Carboxypeptidase | CGGCTACCACTACCGAGAGA | | CCGTAGCTGTGGAAGGTGAG | 165 | |
| Gram-negative bacteria-binding 3-like | GCCTCAACCAGACCTTCAAG | | GAAAGAAGGCGGTATTGCTG | 106 |  |
| Pectin lyase | GTCGGATTCGGCTACAAGAC | | CCAGCCCTCAGAGTTGGTAA | 150 |  |
| Cathepsin B | CATCGGTGAGATCCGTGAC | | CGGTTTCCACCCAGTAGTTC | 209 |  |
| Cytochrome P450 6k1-like | ACAAGTGTGTGAGCGAGACG | | GCCAGTACTTGGGGTCGTAG | 162 |  |
